# Editorial: Advanced computational systems biology approaches for accelerating comprehensive research of the human brain

**DOI:** 10.3389/fgene.2023.1143789

**Published:** 2023-02-09

**Authors:** Zhiwei Ji, Qianqian Song, Jing Su

**Affiliations:** ^1^ College of Artificial Intelligence, Nanjing Agricultural University, Nanjing, China; ^2^ School of Medicine, Wake Forest University, Winston-Salem, NC, United States; ^3^ School of Medicine, Indiana University Bloomington, Bloomington, IN, United States

**Keywords:** systems biology, integrative biology, bioinformatics, multi-omics, human brain

The fast development of various high-throughput sequencing technologies has generated massive valuable human brain atlases (Li and Wang, 2019; Ding et al., 2020), providing great opportunities for systematically understanding the molecular characteristics across different brain regions throughout a series of developmental stages (Parikshak et al., 2015). Analyzing the spatial-temporal characteristics of human brain development and function is of vital importance to determine the causes of a variety of complicated neurological disorders (Bullmore and Sporns, 2009; Parikshak et al., 2015; Khalil et al., 2018; Mirza and Zahid, 2018). Particularly, single-cell sequencing provides a comprehensive landscape of brain cell type diversity (Iourov et al., 2012; Brazovskaja et al., 2019; Mu et al., 2019). However, analyzing such high-dimensional multi-omics data remains substantially complex and thus requires effective and sophisticated computational models and algorithms. Recent progress in computational systems biology fields has facilitated integrative analyses with high precision to obtain new insights into the molecular characteristics of many human diseases (*e.g*., cancers) (Ji et al., 2015; Cossarizza et al., 2019; Ji et al., 2019; Zhao et al., 2021; Ji et al., 2023). However, with the emergence of new Neuro-omics data, developing novel approaches to identify new molecular underpinnings of human brain is still a big challenge (Liu et al., 2018; Li and Wang, 2019; Ji et al., 2021).

This Research Topic entitled “Advanced Computational Systems Biology Approaches for Accelerating Comprehensive Research of the Human Brain” in *Frontiers in Genetics* aims to provide an international forum for.

1) Bringing together the greatest research efforts in brain-specific molecular/network signature identification by integrating multi-omics/multi-level data;
2) Exploring future-generation interesting and practical biomedical applications in AI, machine learning, data sciences, knowledge-based system, etc., to paving the path toward achieving precision medicine in brain disease treatment;
3) Addressing the real-world challenges in the fields of AI-based patient stratification and diagnosis by utilizing advanced machine learning or deep learning, and produce a more reliable and promising application environment to develop those technologies.

Submission for this Research Topic started from May 2022 and closed in October 2022. In nearly 6 months, we received in total 5 paper submissions. All submitted manuscripts had gone through at least three rounds of revision with reviewers in the related fields, including bioinformatics, computational biology, machine learning, and clinical study, etc. The final acceptance rate is 80% with 4 accepted papers in this Research Topic. The summaries of these papers are outlined below.

In the article entitled “Gene Regulatory Identification Based on the Novel Hybrid Time-Delayed Method” by Bao et al. The authors developed a novel two-step GRN inference technique based on the time-delayed correlation coefficient (TDCC) and time-delayed complex-valued S-system model (TDCVSS) to detect the direct associations of GRN more accurately.

In the article entitled “Identification of signaling pathways associated with achaete-scute homology 1 in glioblastomas through Chip-seq data bioinformatics” by Zhang et al. To demonstrate the key role of *ASCL1* gene in the differentiation of neuronal-like glioblastoma (GBM) cancer stem cells, the authors implemented systematic analysis by using Chip-seq data and revealed that *EGFR*, *SPTAN1*, and *CTNN1B* might be the potential down-stream genes of *ASCL1* in GBM development, and *CTNN1B* might make contributions to GBM progression on regulating the *cAMP* pathway.

In the article entitled “Bioinformatics analysis to screen for genes related to myocardial infraction” by Yang et al. The authors integrated multiple gene expression datasets from GEO and implemented deep bioinformatics analysis. Finally, they identified 5 signature genes (*ACOX1, BCL6, CEACAM8, CUGBP2*, *GPX7*) related to Myocardial infarction (MI).

In the article entitled “The study on the morphological changes of oropharynx in patients with complete unilateral cleft lip and palate after palatopharyngeal closure” by Chen et al. The authors mainly focused on investigating the morphology and airway volume of oropharynx patients with unilateral complete cleft lip and palate after palatopharyngeal closure. This study reveals that the airway volume, the minimum cross-sectional area of the pharynx, the horizontal cross-sectional area of the hyoid, and the distance between the hard and soft palate tip in patients with complete unilateral cleft lip and palate show significant differences relative to control samples.

The guest editors would like to thank all the authors submitting their valuable works to this special section of *Frontiers in Genetics*, as well as all peer-reviewers for their great efforts reviewing the submitted articles, providing constructive suggestions and comments and assisting the editors reaching the final decision. Special thanks will be sent to the Editor-In-Chief (EIC), Enrico Domenici, for their precious time and valuable instructions that help us prepare and finalize this Research Topic.
